# Supplementary material for: Proximity labeling of the Tau repeat domain enriches RNA-binding proteins that are altered in Alzheimer’s disease and related tauopathies
Source: bioRxiv. 2025 Jan 22:2025.01.22.633945. Preprint. [Version 1] doi: 10.1101/2025.01.22.633945 (PMC11785194; doi:10.1101/2025.01.22.633945)
Supplement: 2 [file NIHPP2025.01.22.633945v1-supplement-2.pdf]

SUPPLEMENTAL FIGURES

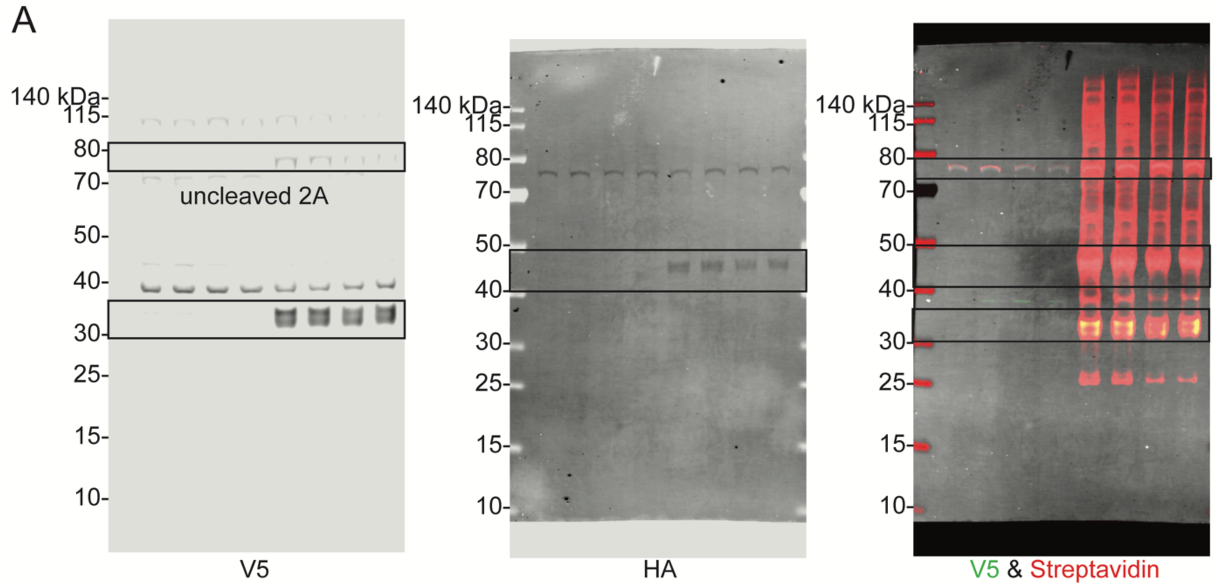

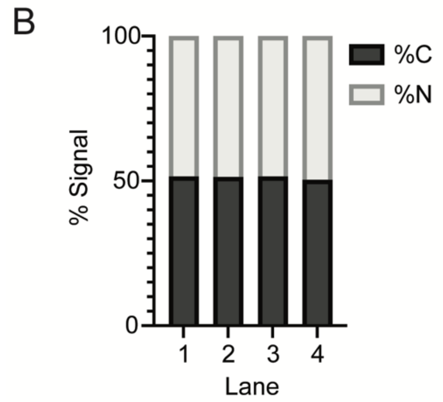

Supplemental Figure S1. 2A efficiently cleaves fragments and equally biotinylates sTurbo TauRD fragments. (A) Cells were transfected with either reagent alone or with sTurbo P301L TauRD 2A plasmid DNA and underwent standard biotin labeling before 8M Urea lysis and protein collection. WB analysis identified recombinant proteins (V5 and HA) and biotinylated proteins. sTurbo TauRD cleavage products were observed at expected molecular weights: ~27 kDa for N-sTurbo TauRD (V5) and ~43 kDa for C-sTurbo TauRD (HA). A faint, uncleaved product (~73 kDa) was observed on the V5 blot. The streptavidin blot also displayed self-biotinylation of the cleaved protein products (HA, white; V5, green). **(B)** Further characterization of the 2A recombinant protein ligase activity reveals the N- and C-sTurbo biotinylated fragments are equally abundant at each ~50% signal via western blot analysis of streptavidin overlaying biochemical tag protein band.

bioRxiv preprint doi: https://doi.org/10.1101/2025.01.22.633945; this version posted January 22, 2025. The copyright holder for this preprint (which was not certified by peer review) is the author/funder, who has granted bioRxiv a license to display the preprint in perpetuity. It is made available under aCC-BY 4.0 International license.

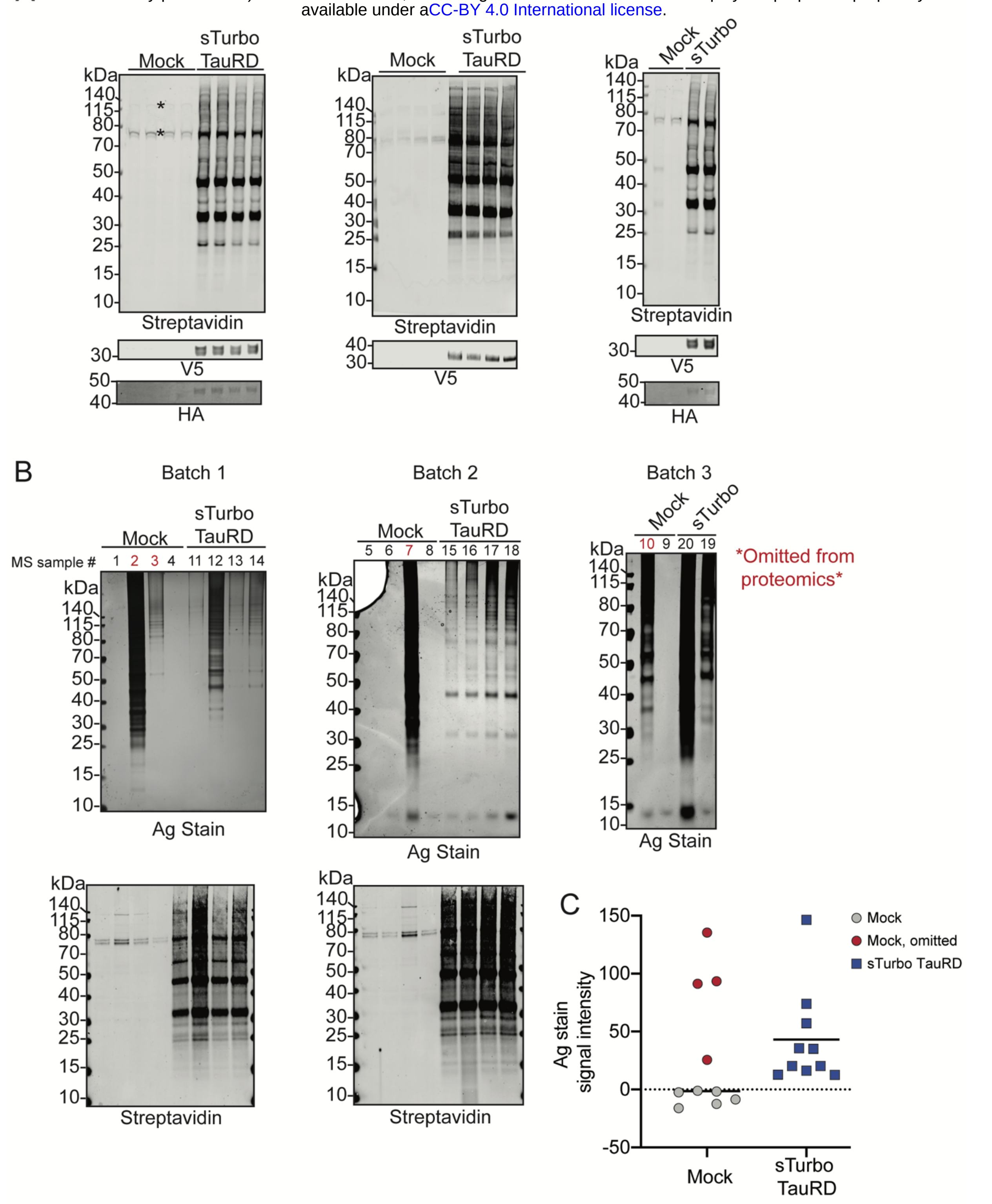

Supplemental Figure S2. Quality control western blots and total protein silver stains. (A) Total lysate input (20 µg) of sTurbo TauRD from HEK293T cells shows robust and consistent labeling across replicates and batches, shown by streptavidin blots. V5 and HA recombinant protein tags were also probed to confirm expression of sTurbo TauRD across lysates. **(B)** Silver stain (Ag stain) displaying total protein after streptavidin affinity purification shows varying total signal in sTurbo TauRD samples and has enriched biotinylated proteins, shown through streptavidin blot. Mock samples with non-specific binding to streptavidin beads, and resulting positive signal found in silver stain, were omitted from this proteomics study (red). Signal intensities from the Ag stain gel lanes are depicted in panel **(C)**.

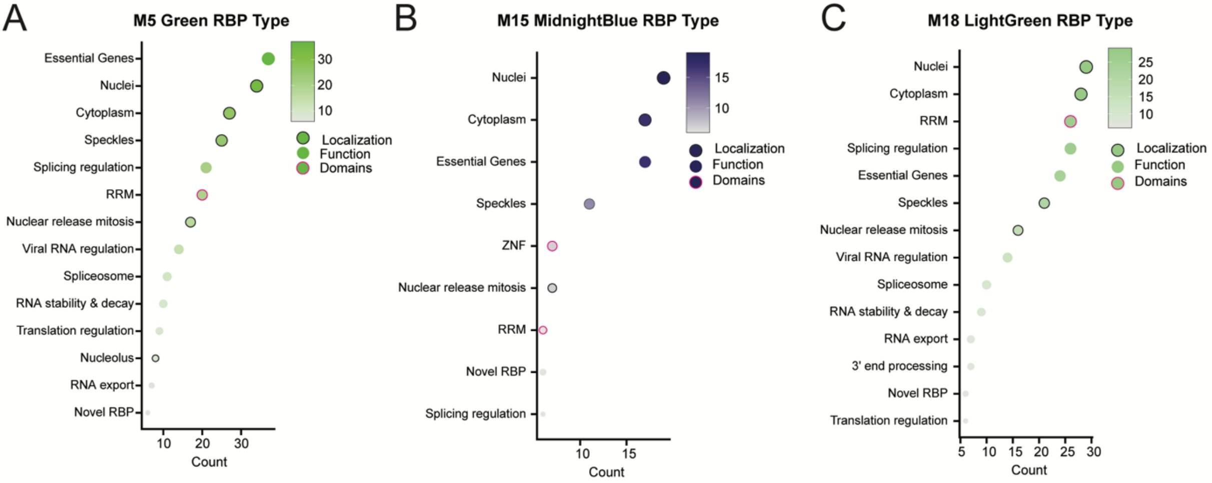

Supplemental Figure S3. Insoluble disease mapping module classification across RBP localization, key functions, and domains. Module members across RBP-enriched modules M5 **(A)**, M15 **(B)**, and M18 **(C)** were integrated with a list of 356 well-characterized RBPs to delineate common and divergent features across each module. Count of module RBPs mapping to each characteristic is visualized.

available under aCC-BY 4.0 International license.

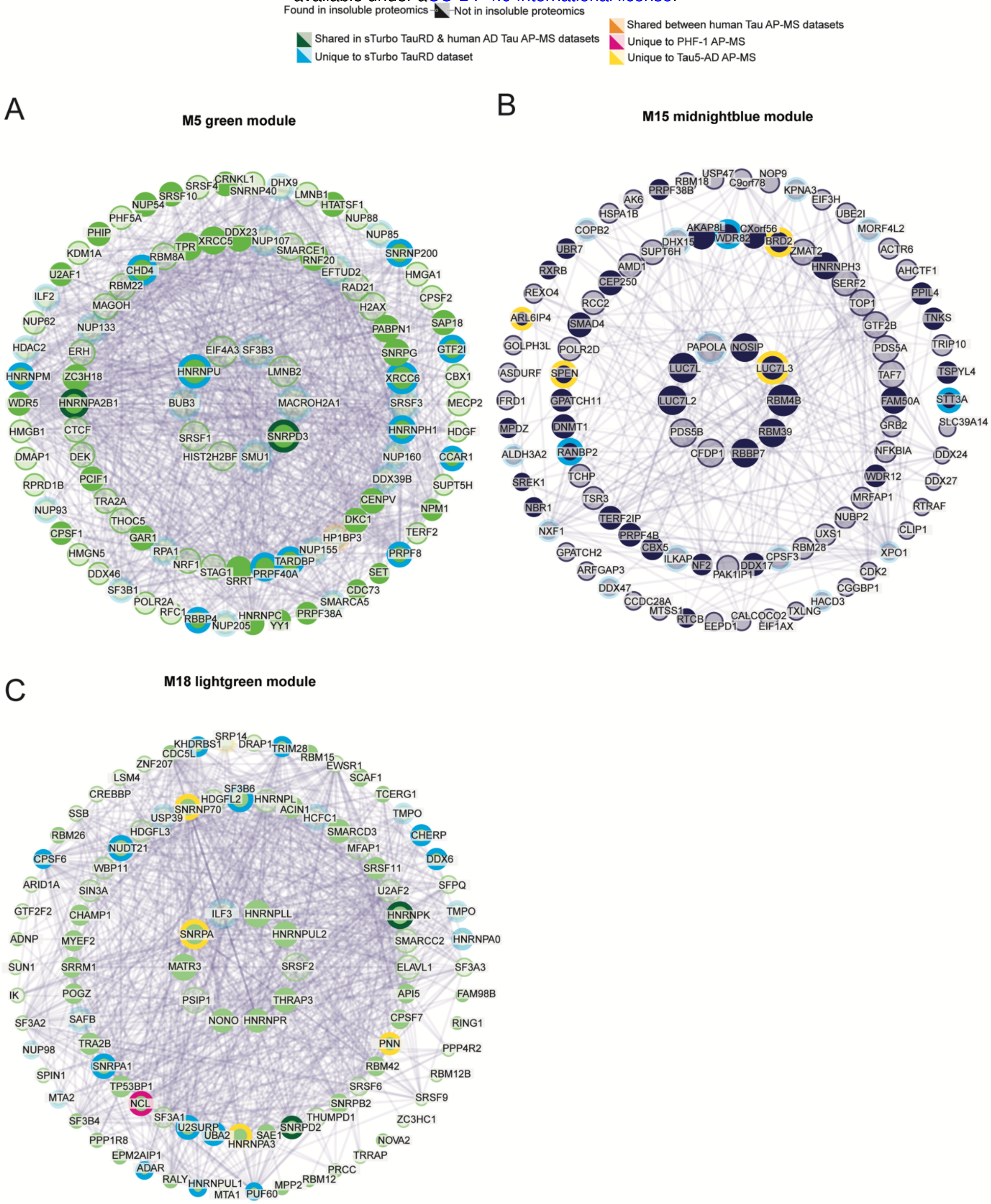

Supplemental Figure S4. Insoluble and RBP-enriched module hub proteins and BioGrid interaction networks. Biogrid integration in which edge lines provide information on biological interactions across hub proteins for insoluble disease-related modules, M18 **(A)**, M5 **(B)** and M15 **(C).** Protein nodes are colored if Tau interactome proteins are identified within the module, either from sTurbo TauRD (unique, blue; shared with AD Tau AP-MS, green) or only described in human Tau AD AP-MS interactomes (warm colors). sTurbo TauRD uniquely captures hub proteins across all three modules, including ILF3, MACROH2A1, SF3B6, and PAPOLA. Shared proteins across Tau interactome datasets include RBPs HNRNPK, SNRPD2, HNRNPA2B1.
